# Supplementary figures and images for: Computational design of Periplasmic binding protein biosensors guided by molecular dynamics
Source: PLoS Comput Biol. 2024 Jun 17;20(6):e1012212. doi: 10.1371/journal.pcbi.1012212 (PMC11213343; doi:10.1371/journal.pcbi.1012212)

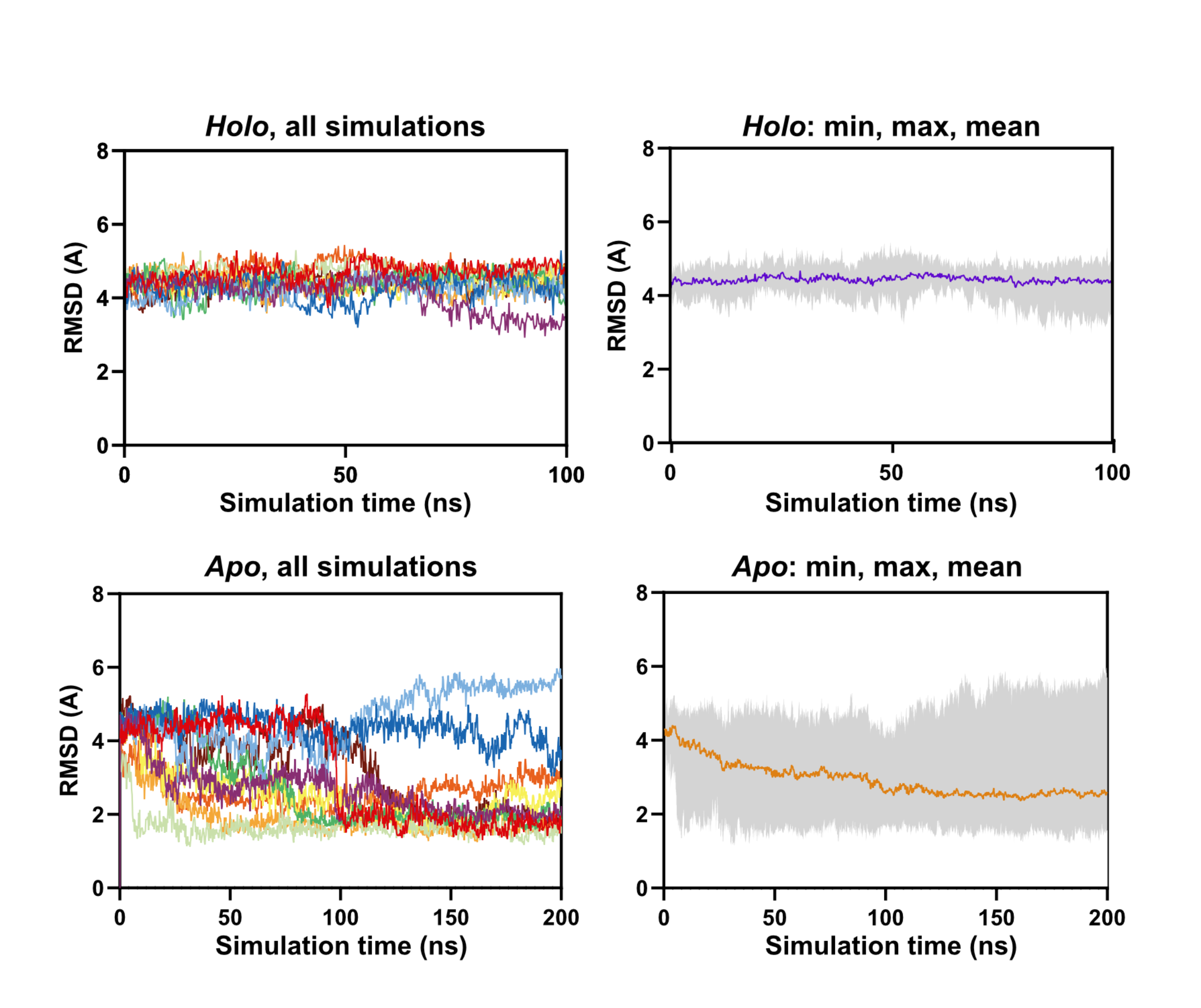

Supplement: S1 Fig — (TIFF) [file pcbi.1012212.s001.tiff]

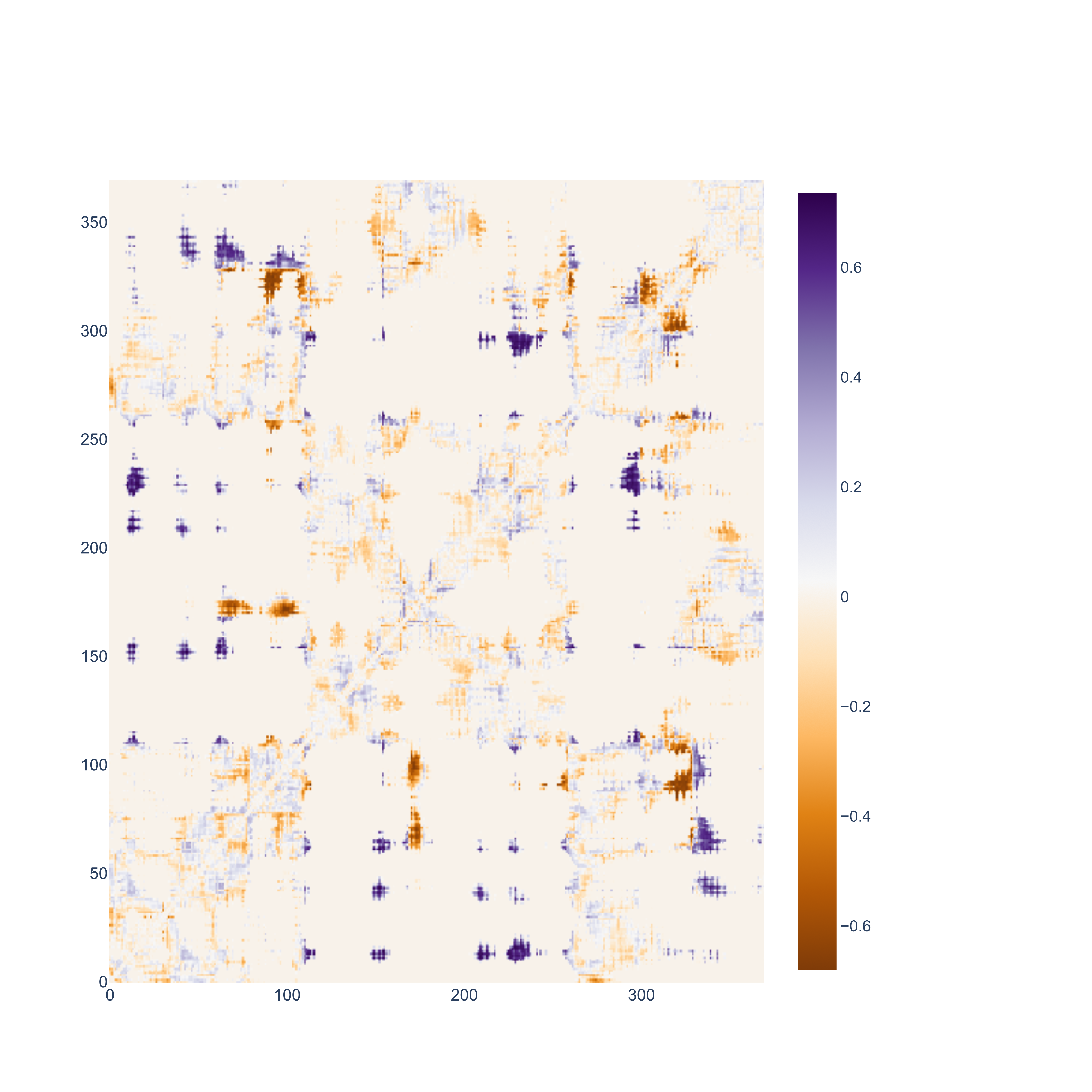

Supplement: S2 Fig — (TIFF) [file pcbi.1012212.s002.tiff]

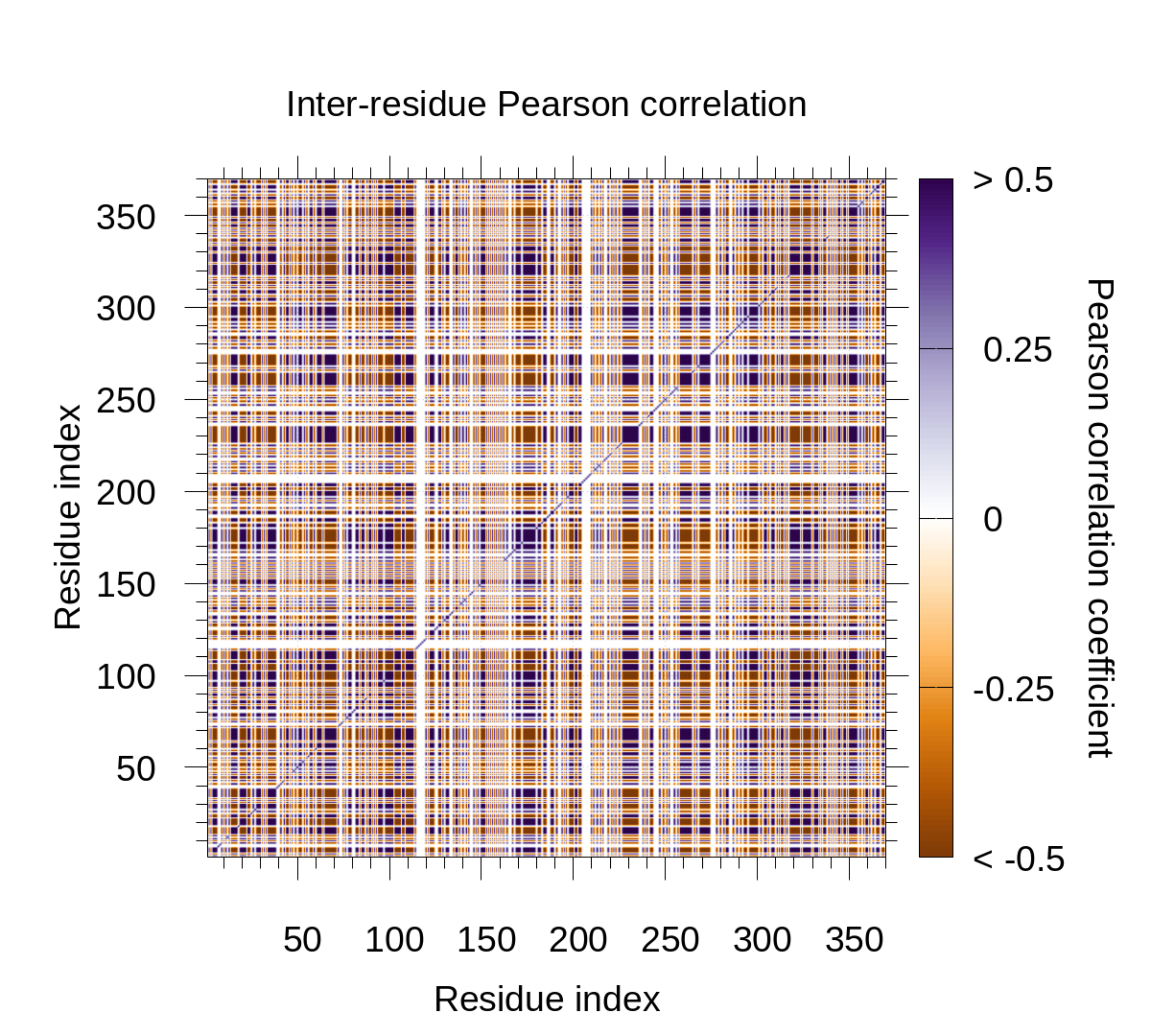

Supplement: S3 Fig — (TIFF) [file pcbi.1012212.s003.tiff]
