# Supplementary material for: Computational design of Periplasmic binding protein biosensors guided by molecular dynamics
Source: PLoS Comput Biol. 2024 Jun 17;20(6):e1012212. doi: 10.1371/journal.pcbi.1012212 (PMC11213343; doi:10.1371/journal.pcbi.1012212)
Supplement: Attachment — Submitted filename: response_to_reviewers.docx [file pcbi.1012212.s007.docx]

Reviewer #1

*1. On page 8, the author writes "we ran 10 x 200 ns of these apo simulations, observing closed-to-open transition in 8/10 of them,". It is a bit vague what criteria authors have used to make this decision. Please provide a quantitative description to support this statement.*

We have addressed this in the manuscript, making it clear that the open and closed states are defined by mean RMSD over the last 50 ns of the simulations.

*2. In Figure 2, panel E, caption, the authors write that they show a "representative" model for open or closed state. How the model was selected, please provide a quantitative description.*

We have made clear in the manuscript that the representative models are those that are close in RMSD to the mean RMSD of the closed and open states respectively.

*3. In figure 2, authors show RMSD compared to the starting structure. How does the RMSD looks like compared to the open state?*

We have added a supplementary figure of the plot of RMSD compared to the open-state crystal structure.

*4. The manuscript seems to be missing a "Conclusion section". Please provide one.*

We have added a “conclusion” heading.

Reviewer #2: The paper suggests that residue position can add to the insights from backbone dihedral angles to generate additional PBP-based biosensors. However, the paper’s major data point is suspect, and the presentation needs improvement.

Experiments: one week’s experiments would probably help. Especially for residue 230—the star of the paper-- the paper’s point is suspect because (a) the counts are so small, and (b) the only increase measured occurs at 100 mM—roughly 100-fold greater than precited from Ref. 10. Because 230 is in the binding pocket for maltose (again, ref (10)), one suspects that mutating this residue violates the authors’ “agnostic” principle—which was never intended to extend to the substrate binding site. I suspect that authors have constructed a defective binding site. It will be necessary to purify the protein—a simple task if the construct includes a His6 sequence. A full dose-response relation should follow.

We see that Reviewer 2’s suggestion would be important to address if residue 230 were the star of the paper, but it was not our intention to lead readers to believe that this is the case. Instead, we intended the “star” of our paper to be the methodology employed to discover the described sensors. We’d like to emphasise that the method didn’t just identify residue 230, it also identified all of the major insertion sites that have been identified previously. As a result, we do not believe that this experiment is necessary, as would not change the conclusion of the paper. However, to avoid misleading readers into believing that the insertion at residue 230 should be considered the main achievement of the paper, we have altered the discussion to focus more on the methodology employed.

Explanations:

The paper ought to better explain the calculations that underlie the suggestions for experiments. reader The final intelligible sentences is ‘We defined a “contact” . . . .’ After that, the become mysterious to the non-cognoscenti. The mysterious concepts include “pixel”, “pixel clustering”, and the residue-level metrics being correlated with each other. Are you simply asking which residues move together (positive) and which move separately? It would be good to say so. GITHUB is not peer-reviewed, so we don’t whether its explanations are clear.

We thank the reviewer for pointing out these omissions. We originally included only a brief explanation the definition of “contact” in the main body of the paper, with another explanation in the methods section. We have added further detail on contact definition and an explanation of clustering to the main body.

It would also be good to state the hardware used to perform he simulations and to analyze the data, so the reader knows whether (in 2024) to perform the analyses using a phone, a tablet, a laptop, a desktop, or a supercomputer.

We have added a description of the hardware used to the methods section.

Additional confusion: the matrix of 3D is symmetrical, but the figure does not state this and shades only along the y-axis. And the correlation color map is puzzlingly congruent with the Y-axis. If the authors give though to the poor reader, this would solve many of the paper’s problems in presentation.Some of the paper’s puzzling features would improve if the experimentalists and computationalists simply talk with each other. Thus, what some of the authors call “raw fluorescence”, others call F0. What one set of authors called “uptick” another set would term, “dose-response relation”.

We have taken onboard Reviewer 2’s suggestions to improve the matrix plot in figure 3, adding a diagonal line to indicate symmetry and modifying the legend to make it clearer.

Pedantic corrections:

“The Pearson Correlation Coefficient matrix can be accessed from only the holo crystal structure,”. Is a very confusing statement, in part because “only” has many interpretations. Perhaps it means, “Knowledge the holo structure is sufficient to begin MD simulations, without the necessity for knowing the apo structure.”

Here is the correct capitalization: Person correlation coefficient. Pearson is a proper noun; his correlation coefficient is not. Although one might capitalize the abbreviation (PCP), one does not capitalize the individual words.

We have addressed these concerns.

Have the authors made all data and (if applicable) computational code underlying the findings in their manuscript fully available?

The PLOS Data policy requires authors to make all data and code underlying the findings described in their manuscript fully available without restriction, with rare exception (please refer to the Data Availability Statement in the manuscript PDF file). The data and code should be provided as part of the manuscript or its supporting information, or deposited to a public repository. For example, in addition to summary statistics, the data points behind means, medians and variance measures should be available. If there are restrictions on publicly sharing data or code —e.g. participant privacy or use of data from a third party—those must be specified.

Reviewer #1: No: Please upload the raw simulation trajectories to any open-source data sharing services.

The full simulation trajectories are multiple gigabytes in size, making them difficult to share. However, we have addressed this in a few ways: 1) We will provide 100 frame trajectories on the GitHub repository associated with the paper. 2) We have performed the simulations in such a way that they are easy to reproduce and have provided all the scripts necessary to do this. 3) We are happy to provide the full trajectories on request and have added a statement to the paper expressing this.

Reviewer #2: Yes
