# Supplementary material for: Computational design of Periplasmic binding protein biosensors guided by molecular dynamics
Source: PLoS Comput Biol. 2024 Jun 17;20(6):e1012212. doi: 10.1371/journal.pcbi.1012212 (PMC11213343; doi:10.1371/journal.pcbi.1012212)
Supplement: Attachment — Submitted filename: Reviewers round 2 comments commented.docx [file pcbi.1012212.s008.docx]

**Comments to the Authors:
Please note here if the review is uploaded as an attachment.**

Reviewer #1: The authors have fully addressed the concerned raised.

Reviewer #2: This critique repeats some of my original comments. Please take additional care to respond to these comments. Part of our profession is efficient communication. At present, some aspects require the reader to work unnecessarily hard.

Figure 3D still needs work. At present, it’s a square. It should be simply an equilateral right triangle (the lower right half of the present square). The suggested tactic would eliminate the redundant, confusing part. The suggested tactic would eliminate the necessity to explain (to stupid readers like me) why the shaded vertical bars are not also shaded horizontal bars. Again, the fact that the color lookup table has the same height as the vertical axis is also confusing. Please make the color lookup table smaller than the vertical axis, so that harried readers (like myself) don’t have to ask whether the lookup table encodes the vertical axis (it does not!).

**We have changed figure 3D as per the reviewer’s suggestion, removing half of the matrix plot and repositioning the legend.**

Th Journal’s web site does not allow me to view the supplementary material. Therefore, I cannot tell whether the authors have rectified the “F0” vs “raw fluorescence” terminology.

**We have made it clearer that “F0” only refers to the “raw fluorescence at 0 mM” and have only used it in context of “dF/F0” to avoid confusion.**

FWIW, what’s the cost of the computer time, the time awaiting intermediate steps in the computational pipeline, and the like? Compared with the cost of time and materials for one round of site-saturated mutagenesis? Is this comparison not even worth considering?

**This would depend very much on the expertise and available resources of the group performing the work. Our study shows that computational design can aid rational design of PBP-based biosensors, and therefore opens the possibility for computational labs to attempt design where before they would never have. Furthermore, the study’s main message is its insights into the underlying reasons for the success of certain designs, in a way that would not be possible, or at least very difficult, in the wet lab. Ultimately, computational studies like this can help target experiments and rationalise results.**
